# A Micro-Quantitative and FFPE-Compatible Workflow for Immunohistochemistry-Guided Spatial Proteomic Analysis of Cellular Subpopulations Within the Tumor Microenvironment

**DOI:** 10.3390/bioengineering13060678

**Published:** 2026-06-11

**Authors:** Junya Peng, Lu Ping, Ruikang Dun, Lulu Liu, Yihong Shi, Ruizhe He, Qing Zhong, Yang Chen, Wenmin Tian, Yupei Zhao

**Affiliations:** 1 State Key Laboratory of Complex and Severe and Rare Diseases, Peking Union Medical College Hospital, Chinese Academy of Medical Science and Peking Union Medical College, Beijing 102602, China; 2 Institute of Clinical Medicine, Peking Union Medical College Hospital, Chinese Academy of Medical Science and Peking Union Medical College, Beijing 100730, China; 3 Department of General Surgery, Peking Union Medical College Hospital, Chinese Academy of Medical Science and Peking Union Medical College, Beijing 100730, China; 4 8-Year MD Program, Chinese Academy of Medical Science and Peking Union Medical College, Beijing 100730, China; 5 Department of Basic Medical Sciences, School of Medicine, Tsinghua University, Beijing 100084, China; 6 Center for Precision Medicine Multi-Omics Research, Institute of Advanced Clinical Medicine, Peking University, Beijing 100080, China; 7 Beijing Advanced Center of Cellular Homeostasis and Aging-Related Diseases, Institute of Advanced Clinical Medicine, Peking University, Beijing 100080, China

**Keywords:** laser microdissection, immunohistochemistry, FFPE tissue, tumor microenvironment

## Abstract

Understanding the spatial proteomic landscape of human tumors is essential for dissecting cellular heterogeneity and microenvironmental interactions in cancer biology. Traditional bulk proteomic approaches, however, obscure spatial information and average out signals from distinct cell populations. Here, we present a detailed and reproducible micro-quantitative protocol for spatially resolved proteomic analysis of specific cellular subpopulations isolated from immunohistochemistry (IHC)-labeled formalin-fixed paraffin-embedded (FFPE) tissue sections using laser microdissection (LMD). By combining IHC staining to visually define phenotypically distinct cells within preserved tissue architecture and precise LMD capture, approximately 6000 target cells can be isolated per sample for downstream proteomic quantification. Despite the ultra-low input, optimized lysis and digestion steps ensure consistent peptide recovery and highly reproducible label-free LC–MS/MS data across replicates. Integrating immunohistochemistry staining-guided spatial sampling with ultrasensitive quantitative proteomics, this workflow enables reliable cell-type-specific profiling directly within human tumor tissues. The protocol bridges histopathology and proteomics, offering a practical framework for translational research exploring spatial protein signatures and tumor microenvironmental heterogeneity.

## 1. Introduction

Spatially resolved proteomics has emerged as a transformative approach, enabling high-definition mapping of protein expression across tissue architecture to delineate the molecular landscape of human tumors at cellular resolution [1]. In cancer biology, tumorigenesis and therapeutic resistance are orchestrated by dynamic interactions between malignant cells and the surrounding stromal and immune components within the tumor microenvironment (TME) [2,3,4]. Conventional bulk proteomic analyses, although highly quantitative, inherently obscure spatial context and average out proteomic signals across heterogeneous cellular populations [5,6]. Consequently, there is an increasing demand for technologies that integrate spatial localization, cell-type specificity, and quantitative proteomic depth within intact tissue specimens—a gap that the present protocol aims to bridge.

Recent advances in laser microdissection (LMD) and mass spectrometry (MS)-based proteomics have enabled high-resolution analysis of spatially defined tissue regions, even at the level of single cells [7,8]. Frameworks such as Deep Visual Proteomics (DVP) exemplify the power of integrating imaging, automated segmentation, and ultra-sensitive MS workflows to capture proteomic heterogeneity with unprecedented depth [7,9]. However, these highly integrated systems typically rely on customized instrumentation, complex software coordination, and expert-level operation. As a result, their implementation remains limited to a small number of specialized laboratories. In most research and diagnostic settings, achieving comparable analytical performance requires workflows that emphasize reproducibility, technical stability, and accessibility—factors that remain key challenges for routine spatial proteomic applications [1,10,11].

In light of these challenges, we sought to establish a broadly applicable and technically stable workflow that enables spatially resolved proteomic analysis from histologically defined regions within routine clinical specimens. Building upon advances in immunohistochemistry-guided laser microdissection and ultrasensitive mass spectrometry, we optimized a micro-quantitative approach compatible with formalin-fixed paraffin-embedded (FFPE) tissues. This workflow enables robust proteomic profiling from microscopic regions containing as few as 6000 cells, with a total experimental turnaround time of approximately three working days from sectioning to quantitative data acquisition.

In this study, we provide a comprehensive, step-by-step guide covering (1) immunohistochemistry staining and visualization of FFPE sections, (2) laser microdissection of defined regions of interest (ROIs), (3) protein extraction and enzymatic digestion under low-input conditions, and (4) label-free LC–MS/MS quantification. Together, these modules provide a robust and accessible framework that bridges histopathology and spatial proteomics, empowering the quantitative dissection of cell-type-specific proteomic landscapes within the tumor microenvironment.

## 2. Materials and Methods

### 2.1. Immunohistochemistry (IHC) Staining of FFPE Sections

**Tissue sectioning and slide preparation**

Formalin-fixed paraffin-embedded (FFPE) tumor tissues were sectioned at a thickness of 10 μm using a rotary microtome (Microme HM340E, Thermo Fisher Scientific, Waltham, MA, USA). Sections were mounted onto polyethylene naphthalate (PEN) membrane-coated, RNAse-free slides (50102, Gene Company Limited, Hong Kong, China) specifically designed for subsequent laser microdissection (LMD). The slides were tilted and air-dried at room temperature for 60 min, followed by UV exposure for 30 min to ensure tight adhesion between the tissue and the PEN membrane.

**Deparaffinization and rehydration**

Slides were deparaffinized by sequential immersion in xylene-free deparaffinization solutions (YA0031, Solarbio, Beijing, China) for 10 min each. Sections were then rehydrated through a graded ethanol series (100%, 95%, 90%, 80%, 70%; 5 min each) and rinsed in distilled water for 5 min to complete rehydration.

**Heat-induced epitope retrieval (HIER)**

Antigen retrieval was carried out in 10 mM citrate buffer (pH 6.0) prepared with citrate buffer tablets (ZLI-9064, Zhong Shan-Golden Bridge Biological Technology Co., Ltd., Beijing, China) using a microwave oven (Midea). Slides were heated at 100 W for 3 min, followed by 30 W for 10–15 min to sustain gentle boiling. After retrieval, slides were allowed to cool naturally to room temperature and washed three times with PBS (pH 7.4, 5 min each) to remove residual buffer salts.

**Blocking and antibody incubation**

To reduce nonspecific binding, tissue sections were incubated in blocking buffer containing 10% fetal bovine serum (FBS; A5669701, Gibco, Thermo Scientific, Waltham, MA, USA) and 0.1% saponin (S33760, Acmec, Shanghai, China) in PBS for 30 min at room temperature.

Primary antibody incubation was performed at room temperature for 2 h in the same blocking buffer using anti-anterior gradient 2 (AGR2; secretory lineage marker; ab76473, Abcam, Cambridge, CB2 0AX, UK; dilution 1:200)

After primary incubation, slides were washed three times with PBS (5 min each) and subsequently incubated with goat anti-rabbit IgG (H&L)-HRP conjugated secondary antibody (BE0101-100, Bioeasy, Beijing, China) diluted in the same blocking buffer containing 10% fetal bovine serum and 0.1% saponin in PBS at room temperature for 1 h.

After secondary incubation, slides were rinsed three times with PBS (5 min each). Slides were stained with DAB (ZLI-9018, Zhong Shan-Golden Bridge Biological Technology Co., Ltd., Beijing, China) for 5 min. After rinsing with PBS, slides were subsequently stained with Hematoxylin (G1140, Solarbio, Beijing, China). Dehydration and sealing were not performed and the slides were air-dried before LMD.

### 2.2. Laser Microdissection (LMD) of Defined Regions of Interest (ROIs)

**Overview and instrument setup**

Laser microdissection (LMD) was performed using a CellCut system (MMI, Gene Company Limited, Hong Kong, China) equipped with a 355 nm laser module and an automated stage. Before dissection, all optical components were cleaned with lens-grade ethanol (10009218, Sinopharm, Beijing, China), and the laser focus was calibrated according to the manufacturer’s instructions. PEN-membrane slides containing immunohistochemistry (IHC)-stained sections were placed on the stage, and the tissue orientation was adjusted to match the corresponding digital images acquired during IHC imaging.

**Region identification and ROI annotation**

Signals from marker channels (e.g., AGR2) were used to identify distinct cellular subpopulations within the tumor microenvironment. Using the LMD control software (MMI CellTools, Version 6.0), regions of interest (ROIs) were delineated based on:

(1) Morphological integrity (clear cell boundaries, preserved architecture);
(2) Marker expression pattern (AGR2^+^ or AGR2^−^ tumor regions, or other IHC-defined subsets);
(3) Exclusion of artifacts (folded tissue, necrotic areas, debris).

ROIs were manually outlined under a 20× objective. The typical area for each ROI corresponded to approximately 1000–3000 cells, depending on tissue density and section thickness. For each biological sample, two FFPE tissue sections from the same specimen were mounted onto the same PEN membrane slide and independently subjected to IHC staining and ROI annotation. Importantly, the two sections were separated by approximately 10 sectioning intervals rather than being directly adjacent serial sections, thereby minimizing the likelihood of repeatedly sampling the exact same cells.

**Laser cutting and collection parameters**

Dissection was conducted in clean-room conditions to avoid contamination. The following laser parameters were optimized for precise sectioning of FFPE tissues on PEN membranes: Laser power: 6.8 mW; Laser wavelength: 355 nm; Cut velocity: 50 μm/s; Laser focus: 52%; Repeat: 1.

The laser cutting path was defined to enclose each ROI completely without intersecting adjacent tissue. Cut fragments were collected by gravity-assisted fall or adhesive cap capture into 0.5 mL low-bind microcentrifuge tubes (MMI Isolation Caps transparent, 50204).

For each biological replicate, multiple ROIs (typically 3–6 per case) were pooled into a single collection tube to ensure sufficient material for downstream proteomic processing. Multiple ROIs from the two tissue sections were independently microdissected and pooled into the same collection tube for downstream LC–MS/MS analysis. Biological replicates in this study refer to independent patient tumor samples. The collection amount was empirically optimized according to the analytical sensitivity and reproducibility of the LC-MS/MS workflow to ensure stable protein identification across samples. Blank membrane areas adjacent to the tissue were also dissected as negative controls to monitor potential background contamination.

**Post-collection handling**

After completion of LMD, samples were immediately sealed and stored at −80 °C until protein extraction. The dissection stage and laser focus path were cleaned between each sample using sterile cotton swabs soaked in 70% ethanol to prevent cross-sample contamination.

**Quality control**

To verify successful collection, both before- and after-dissection images were recorded using the LMD control software. ROI coordinates and cutting metadata (laser power, time, path length, area) were exported as CSV log files for traceability. Representative images showing clean boundaries and complete excision were archived for documentation. Pre- and post-dissection images were reviewed for each ROI to confirm cutting accuracy and completeness, and regions with incomplete cutting, tissue folding, or suboptimal morphology were excluded from downstream collection. To minimize technical variation, ROIs were collected with comparable areas and identical section thicknesses, thereby maintaining relatively consistent collection volumes across samples. All downstream proteomic analyses were subsequently performed using normalized protein abundance values.

### 2.3. Protein Extraction and Enzymatic Digestion Under Low-Input Conditions

**Lysis buffer preparation and Sample resuspension**

Laser-microdissected tissue fragments collected in low-bind microcentrifuge tubes were lysed in 150 μL of lysis buffer containing 300 mM Tris-HCl pH = 9, 200 mM glycine and 2% SDS. Samples were gently vortexed and briefly centrifuged to ensure that all tissue fragments were submerged in the lysis buffer. To enhance protein solubilization and reverse formalin-induced crosslinks, lysates were incubated at 95 °C for 60 min in a thermomixer (C5382) set to 1000 rpm, followed by cooling down to room temperature. The supernatant was precipitated with 6 volumes of acetone overnight at −20 °C.

**Reduction and alkylation**

The precipitate was resuspended in 20 μL of 8 M Urea (500 mM Tris-HCl, pH 8.5) and sequentially treated with Tris (2-carboxyethyl) phosphine hydrochloride (10 mM) (PG82080, Thermo Scientific, Waltham, MA, USA) and iodoacetamide (25 mM) (I1149, Sigma, St. Louis, MO, USA) at room temperature.

**Protein digestion under low-input conditions**

For proteolytic digestion, approximately 500 ng of Trypsin/Lys-C Mix (V5071, Promega, Madison, WI, USA) was added to each sample. Samples were incubated at 37 °C for 16–18 h with shaking at 1000 rpm in a thermomixer.

Digestion was terminated by adding formic acid (FA) to a final concentration of 0.5% (*v*/*v*). Samples were centrifuged at 14,000× *g* for 10 min at 4 °C, and the peptide-containing supernatants were transferred to low-bind tubes.

**Peptides desalting and concentration**

Peptides were desalted using a Monospin C18 column (GL Sciences, Tokyo, Japan). Columns were conditioned with 100 μL of 100% acetonitrile (ACN, 271004, Sigma, St. Louis, MO, USA), followed by 100 μL of 0.1% FA in water. Samples were loaded onto the columns by centrifugation at 1000× *g* for 5 min. The columns were washed with 100 μL of 0.1% FA in water and eluted with 30 μL of 60% ACN/0.1% FA. The eluates were vacuum-dried at room temperature (7810040, LABCONCO, Kansas, MO, USA) and redissolved with 0.1% FA to a final concentration of 0.2 ng/μL before LC–MS/MS analysis.

**Quality assessment of Peptide yield**

Peptide concentration was not measured due to the low peptide yield (<1 μg) and the risk of sample loss. Instead, the entire peptide sample was loaded for LC-MS/MS analysis. To ensure reliable comparison across samples, normalization was performed on search results.

### 2.4. Label-Free LC-MS/MS Quantification

**Peptide separation by nanoLC**

Peptide mixtures were analyzed using an EASY-nLC 1200 HPLC (Thermo Scientific, Waltham, MA, USA) coupled online to a high-resolution Orbitrap mass spectrometer (Q Exactive HF-X, Thermo Scientific, Waltham, MA, USA). Analytical separation was carried out on a homemade reversed-phase C18 column (75 μm ID × 250 mm, 1.9 μm particles) maintained at 50 °C.

A binary solvent system was employed:

- Solvent A: 0.1% FA in water
- Solvent B: 0.1% FA in 80% acetonitrile (ACN)

The trypsin-digested peptides were separated at a flow rate of 0.3 µL/min using a 135-min linear gradient as follows: mobile phase B was increased from 3% to 8% over 0–2 min, from 8% to 32% over 2–102 min, from 32% to 45% over 102–127 min, and from 45% to 95% over 127–130 min, followed by a hold at 95% B from 130 to 135 min.

**Mass spectrometry data acquisition**

Eluted peptides were ionized using a nano-electrospray ionization (NESI) source with a spray voltage of 2.2 kV and a capillary temperature of 300 °C. The mass spectrometer was operated in data-dependent acquisition (DDA) mode.

Full MS scans: *m*/*z* 350–1500 at resolution 60,000, AGC target 3 × 10^6^, maximum injection time 50 ms.

MS/MS scans: Top 30 most intense ions fragmented by higher-energy collisional dissociation (HCD) at normalized collision energy (NCE) 30, resolution 15,000, AGC target 5 × 10^5^, maximum injection time 40 ms, dynamic exclusion 30 s.

**Raw data processing and peptide identification**

Database searching was performed using Proteome Discoverer (PD, version 2.3, Thermo Scientific, Waltham, MA, USA) with Sequest HT and Percolator.

The processing workflow was PWF_QE_Precursor_Quan_and_LFQ_SequestHT_Percolator, and the consensus workflow was CWF_Comprehensive_Enhanced Annotation_LFQ_and_Precursor_Quan. The key search parameters and thresholds were as follows:

- Protein database: Uniprot human database (reviewed only)
- Enzyme: Trypsin (full specific), maximum missed cleavage sites: 2
- Fixed modification: Carbamidomethyl (C)
- Variable modifications: Oxidation (M), Acetyl (Protein N-term)
- Precursor mass tolerance: 10 ppm
- Fragment mass tolerance: 0.02 Da
- Percolator FDR threshold: q-value ≤ 0.01 (1% FDR) at both peptide and protein levels

The raw data obtained after database searching are provided in Table S1.

### 2.5. Statistical Analysis

OmicsBean software (V1.0, Shanghai, China) was used for proteomic data processing and analysis, including missing value imputation, normalization, and principal component analysis (PCA). Normalization was performed in two sequential steps as follows. First, each column (one sample) was scaled by sum normalization, in which every value was divided by the column sum and multiplied by 1000. Second, a generalized log transformation was applied to every record x in the whole data matrix as follow:

$${log}_{2}[\frac{x+\sqrt{x^{2}+min.{val}^{2}}}{2}]$$

The small offset, min.val, was defined as one-tenth of the minimum absolute value in the data. This generalized logarithm is meant to avoid the singularity of the standard log transform at zero while approximating a base-2 logarithm for the bulk of the data. The normalized data are provided in Table S2. Protein fold-change values were calculated using Genefilter. Differentially expressed proteins were identified using a Log_2_ fold change > 1 and a *p* value < 0.05 by *t* test as the cutoff criteria (Table S3). Heatmap and network visualization were generated using ggplot2 package and Cytoscape v.3.5.129 implemented in the OmicsBean workbench.

Statistical analysises were performed in GraphPad Prism 9.0. Data are presented as the means ± standard error of mean (SEM). Comparison between two groups was performed by two-tailed Student’s *t*-test. *p* < 0.05 was considered statistically significant with *, *p* < 0.05; **, *p* < 0.01; ***, *p* < 0.001 used to denote the levels of significance.

## 3. Results

### 3.1. Workflow Overview

**Figure 1:**
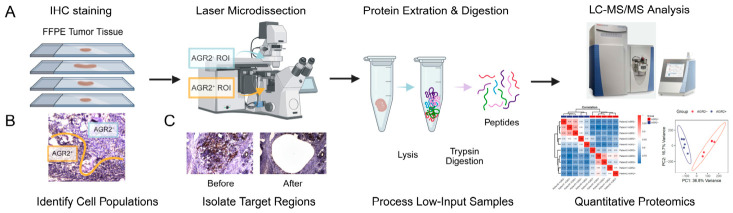
(**A**) Schematic illustration of the four-step workflow showing IHC staining, LMD capture, peptide processing, and LC–MS/MS quantification. (**B**) Representative IHC images showing AGR2 staining patterns. (**C**) Representative pre- and post-LMD micrographs demonstrating clean ROI boundaries and consistent cell counts.

To provide an overview of the established protocol, we designed an integrated workflow combining immunohistochemistry (IHC)–guided laser microdissection (LMD) with low-input LC–MS/MS–based proteomics (Figure 1A). The workflow consists of four sequential modules: (1) Marker-based IHC staining and visualization of FFPE tissue sections to identify phenotypically distinct cell populations; (2) precise LMD capture of histologically defined microscopic regions; (3) micro-quantitative protein extraction and enzymatic digestion optimized for minimal tissue input; and (4) label-free LC–MS/MS quantification followed by bioinformatic analysis.

The entire workflow—from slide preparation to proteomic data acquisition—can be completed within approximately three working days: IHC staining and imaging require about 2–3 h, LMD collection requires about 30–45 min per slide, and peptide processing together with LC–MS/MS analysis requires an additional ~55 h. Subsequent database search and proteomic data processing using Proteome Discoverer (PD) required approximately 5 h of computation time for the complete dataset. This compact workflow ensures rapid turnaround while maintaining compatibility with routine pathology laboratory schedules. Importantly, the workflow is broadly accessible without specialized machine learning or deep learning from high-resolution images prior to laser microdissection.

### 3.2. Validation of IHC-Guided LMD Sampling

To evaluate the precision and reproducibility of the IHC-guided LMD process, immunohistochemistry staining for anterior gradient 2 (AGR2) was performed on FFPE tumor sections. AGR2 staining clearly delineated distinct tumor subpopulations, namely AGR2^+^ and AGR2^−^, within the same histological tumor context, enabling unambiguous identification of phenotypically defined regions of interest (ROIs) (Figure 1B). AGR2 was selected as an example marker in this protocol because previous multi-omics studies in NF-panNETs have identified AGR2-high tumor cells as a biologically distinct subpopulation associated with elevated proliferative signatures, metastasis-like primary (MLP) programs, and poorer clinical outcomes. The detailed biological characterization of AGR2 will be reported elsewhere.

Based on the IHC signal, AGR2^+^ tumor regions (representing AGR2-positive tumor cells) and AGR2^−^ tumor regions (representing AGR2-negative tumor cells) were manually annotated and precisely isolated by LMD from the same FFPE tissue section of each patient, enabling a strictly patient-matched sampling design. Laser cutting cleanly excised the predefined ROIs, as confirmed by direct comparison of pre- and post-dissection images (Figure 1C).

Across five independent FFPE tumor specimens, AGR2^+^ and AGR2^−^ subpopulations were successfully collected as paired samples from each individual tumor. This ensured that both phenotypes were analyzed under identical tissue preservation, staining and microdissection conditions. For each subpopulation, the total dissected area was approximately 12 mm^2^ with a section thickness of 10 μm, corresponding to an estimated ~6000 cells per subpopulation based on nuclear density and section thickness.

This patient-matched sampling strategy was intentionally adopted to minimize interpatient variability and to enable robust intra-tumoral comparisons between AGR2-defined phenotypes. Dissection of all target ROIs on a single slide was typically completed within 30–45 min, supporting the practical feasibility of the workflow. Post-dissection inspection under bright-field microscopy confirmed complete removal of the designated ROIs while preserving the integrity of surrounding tissue, indicating high capture purity and absence of cross-contamination.

### 3.3. Quantitative Reproducibility and Analytical Performance of the Low-Input Workflow

To determine whether the workflow supports reliable proteomic analysis under low-input conditions, we first evaluated peptide recovery and digestion performance from microdissected tumor subpopulations. Using approximately 6000 cells per subpopulation, the optimized lysis and digestion protocol consistently generated homogeneous lysates suitable for LC-MS/MS analysis.

**Figure 2:**
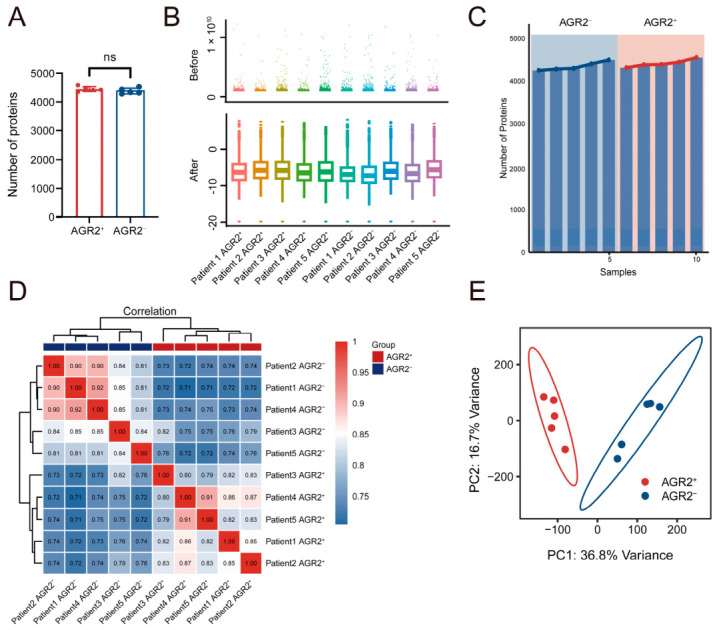
Quantitative reproducibility and analytical performance of the low-input workflow. (**A**) Numbers of proteins identified in AGR2^+^ and AGR2^−^ subpopulations. (**B**) Protein abundance distributions across replicates showing consistent signal intensity and minimal technical variance. (**C**) Number of proteins identified in each proteomic sample. (**D**) Pairwise correlation analysis of protein abundance profiles with unsupervised in hierarchical clustering. (**E**) Principal component analysis (PCA) illustrating separation of AGR2^+^ and AGR2^−^ subpopulations with reproducible clustering within phenotypes.

Building on this, we next assessed the quantitative reproducibility and analytical performance of the workflow. Label-free-LC-MS/MS analysis of AGR2^+^ and AGR2^−^ subpopulations yielded deep and consistent proteomic profiles across patient-matched samples. A total of 4286–4588 proteins were identified per subpopulation, demonstrating stable proteome coverage under microregional sampling conditions (Figure 2A). Global protein abundance distributions were highly comparable across biological replicates, demonstrating consistent signal intensity and minimal technical variation introduced during sample preparation and data acquisition (Figure 2B). Comparable numbers of proteins in each sample were identified across the two subgroups (AGR2^−^ and AGR2^+^), supporting the robustness and the reproducibility in the peptide detection between the two subgroups. (Figure 2C). Pairwise correlation analysis of protein abundance profiles further confirmed high reproducibility across samples. Within each phenotype, AGR2^+^ and AGR2^−^ subpopulations showed strong correlations, whereas lower correlation was observed between phenotypes, indicating distinct proteomic profiles. Unsupervised hierarchical clustering based on correlation distances further separated AGR2^+^ and AGR2^−^ samples into two distinct groups (Figure 2D). Consistently, principal component analysis (PCA) demonstrated clear separation between AGR2^+^ and AGR2^−^ tumor subpopulations, while maintaining tight clustering within each phenotype across replicates, indicating high quantitative consistency and preservation of biological differences (Figure 2E).

Together, these results demonstrate that the workflow enables robust, reproducible, and quantitatively reliable proteomic profiling from low-input, spatially defined tumor regions.

### 3.4. Distinct Proteomic Signatures of Cell Subpopulations

**Figure 3:**
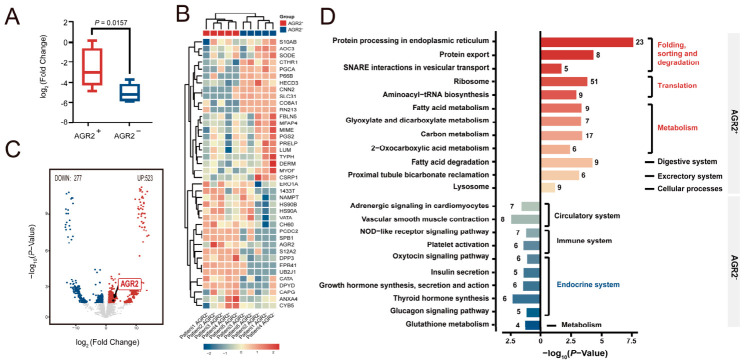
Distinct proteomic signatures of AGR2^+^ and AGR2^−^ tumor subpopulations. (**A**) Relative AGR2 protein abundance in AGR2^+^ and matched AGR2^−^ tumor regions. (**B**) Heatmap of differentially expressed proteins showing consistent separation between subpopulations across five specimens. (**C**) Volcano plot of 800 significant proteins (*p* < 0.05, Log_2_ fold change > 1). (**D**) KEGG enrichment map highlighting pathways upregulated in each phenotype.

Having established the quantitative reproducibility and analytical stability of the workflow, we next assessed whether it could resolve biologically distinct tumor subpopulations defined by immunohistochemistry-guided sampling. As a direct validation of sampling specificity, AGR2 protein abundance was consistently higher in AGR2^+^ microdissected regions than in matched AGR2^−^ regions across all five patient pairs, confirming accurate proteomic capture of the intended phenotypic compartments (Figure 3A).

Differential proteomic analysis identified a set of proteins that were consistently altered between AGR2^+^ and AGR2^−^ subpopulations. A substantial fraction of these proteins showed concordant log_2_fold-change directions across individual patient pairs, supporting the robustness and cross-patient stability of the observed differences (Figure 3B).

Using paired statistical analysis (*p* < 0.05, Log_2_ fold change > 1, we identified approximately 800 proteins that differed significantly in abundance between AGR2^+^ and AGR2^−^ subpopulations. Visualization by volcano plot revealed well-defined distributions of up- and down-regulated proteins, consistent with systematic biological divergence rather than stochastic variation (Figure 3C).

Functional enrichment analysis based on KEGG pathways revealed distinct pathway-level programs between AGR2^+^ and AGR2^−^ tumor subpopulations (Figure 3D). At the global level, AGR2^+^ regions were primarily enriched in pathways associated with protein processing, translation, and metabolism, including protein processing in the endoplasmic reticulum, ribosome, aminoacyl-tRNA biosynthesis, and multiple metabolic pathways such as carbon metabolism and fatty acid metabolism. Functionally, these pathways converge on enhanced protein synthesis, folding, and trafficking capacity, coupled with increased metabolic support, collectively reflecting a highly biosynthetically active and secretion-competent cellular state that is consistent with the previously observed proliferative and malignant features of AGR2^+^ tumor cells (doi: 10.1016/j.scib.2026.06.017).

In contrast, AGR2^−^ subpopulations were enriched in endocrine- and signaling-related pathways, including insulin secretion, glucagon signaling pathway, Growth hormone synthesis, secretion and action, and Thyroid hormone synthesis, indicating retention of differentiated endocrine functions (Figure 3D).

Pathway-level heatmaps of representative KEGG pathways further supported these findings by demonstrating consistent regulation of pathway-associated proteins within AGR2^+^ and AGR2^−^ subpopulations across patient-matched samples (Figure S1).

Collectively, these findings demonstrate that the established workflow is capable of resolving biologically meaningful proteomic differences between spatially defined tumor subpopulations under low-input conditions. These results support the applicability of the workflow for dissecting cell-type-specific functional heterogeneity within the tumor microenvironment.

## 4. Discussion

In this study, we established a practical and reproducible workflow for spatially resolved proteomic analysis of histologically defined cellular subpopulations within FFPE tumor tissues. By integrating immunohistochemistry-guided laser microdissection with low-input LC-MS/MS quantification, this workflow enables robust proteomic profiling from microscale regions containing only a limited number of cells. Importantly, the protocol is compatible with routine FFPE pathology specimens and standard laboratory infrastructure, supporting broader implementation of spatial proteomic analysis in translational and clinical research settings.

A major advantage of the current workflow lies in its balance between spatial specificity, technical robustness, and practical accessibility. Other spatial proteomic and multiplexed imaging approaches, including Imaging Mass Cytometry (IMC), cyclic immunofluorescence (cycIF), and CODEX/PhenoCycler, provide powerful high-dimensional spatial characterization capabilities but generally depend on predefined and validated antibody panels, thereby limiting analysis to selected targets [12,13,14,15]. Deep Visual Proteomics (DVP) further enables ultra-high-resolution spatial proteomic analysis but relies on highly integrated instrumentation, automated imaging-analysis pipelines, and substantial technical expertise [7,9]. Although several previous studies have combined laser microdissection with proteomic analysis, relatively few have provided detailed and reproducible workflows specifically designed for immunohistochemistry-guided spatial proteomics in FFPE tissues [16]. For example, a recent LMD-based proteomic study of paraffin-embedded pediatric and fetal lung tissues primarily relied on anatomically defined tissue regions, such as bronchioles and alveoli, for spatial sampling [17]. In contrast, the current IHC-guided LMD workflow combines immunohistochemistry-defined phenotypic labeling with downstream LC–MS/MS-based proteome-wide analysis, enabling broader exploration of proteins and pathways within biologically distinct tumor subpopulations directly within preserved FFPE tissue architecture. In addition, the current workflow achieved reproducible quantification of more than 4500 proteins from low-input FFPE-derived samples while providing a detailed step-by-step protocol covering tissue processing, ROI collection, peptide preparation, and quantitative analysis. Compared with DVP-based approaches that require machine learning-assisted segmentation strategies prior to laser microdissection, the present workflow is technically more straightforward and remains compatible with routine pathology laboratory workflows, thereby potentially facilitating broader implementation in translational and clinical research settings.

Beyond technical considerations, this workflow also demonstrated its capability to capture biologically meaningful proteomic differences between spatially defined tumor cell states. Using AGR2 as a representative marker, we identified distinct proteomic programs between AGR2^+^ and AGR2^−^ tumor regions within the same tissue context. AGR2^+^ regions were enriched in pathways associated with protein synthesis, endoplasmic reticulum protein processing, ribosome activity, and cellular metabolism, whereas AGR2-regions retained endocrine-related signaling features. Although the detailed biological characterization of AGR2-associated tumor states will be reported separately, these findings support the ability of the workflow to capture biologically relevant spatial heterogeneity directly from preserved clinical tissues.

Several limitations of the current workflow should be considered. First, although the method enables analysis from low-input material, the requirement for pooling multiple microdissected regions per sample limits its resolution compared to true single-cell proteomic approaches. Second, the LMD process relies on manual ROI selection, which may introduce operator-dependent variability and constrain throughput in large-scale studies. Third, the use of FFPE tissues, while clinically advantageous, imposes inherent challenges due to protein crosslinking and potential loss of certain protein species. In addition, as the current study was performed in a relatively small pilot cohort, the present findings should primarily be interpreted as a proof-of-concept methodological demonstration rather than large-scale biological validation. Further evaluation in larger independent cohorts and broader validation settings will be important to assess the generalizability and robustness of the workflow.

Future developments may further enhance the utility of this workflow. Integration with spatial transcriptomics or multiplexed imaging modalities could enable multi-omic characterization of tumor ecosystems [18]. Advances in automated image analysis and ROI selection may improve throughput and reduce operator bias. In addition, continued optimization of sample preparation and mass spectrometry sensitivity may further reduce input requirements, moving toward higher-resolution spatial proteomics.

In summary, we provide an accessible and robust framework for spatially resolved proteomic analysis in routine clinical specimens. By combining histopathological guidance with quantitative proteomics, this workflow offers a practical approach for investigating cell-type–specific protein expression and functional heterogeneity within the tumor microenvironment, with broad applicability in both basic research and translational studies.

## Data Availability

The original data presented in the study are openly available in the ProteomeXchange Consortium via the iProX partner repository with the dataset identifier PXD078338.

## Supplementary Materials

The following supporting information can be downloaded at: https://www.mdpi.com/article/10.3390/bioengineering13060678/s1, Figure S1: Pathway-level heatmaps of representative KEGG pathways in AGR2^+^ and AGR2^−^ in tumor subpopulations; Table S1: Raw data; Table S2: Normalization; Table S3: Differentially Expressed Proteins.

## Abbreviations

The following abbreviations are used in this manuscript:

AGR2^+^	AGR2-positive
AGR2^−^	AGR2-negative
