# Supplementary material for: A Micro-Quantitative and FFPE-Compatible Workflow for Immunohistochemistry-Guided Spatial Proteomic Analysis of Cellular Subpopulations Within the Tumor Microenvironment
Source: Bioengineering (Basel). 2026 Jun 11;13(6):678. doi: 10.3390/bioengineering13060678 (PMC13296145; doi:10.3390/bioengineering13060678)
Supplement: Supplementary file 1 [file bioengineering-13-00678-s001.zip › bioengineering-4297113-supplementary/Supplementary Figure & Tables/Figure S1.pdf]

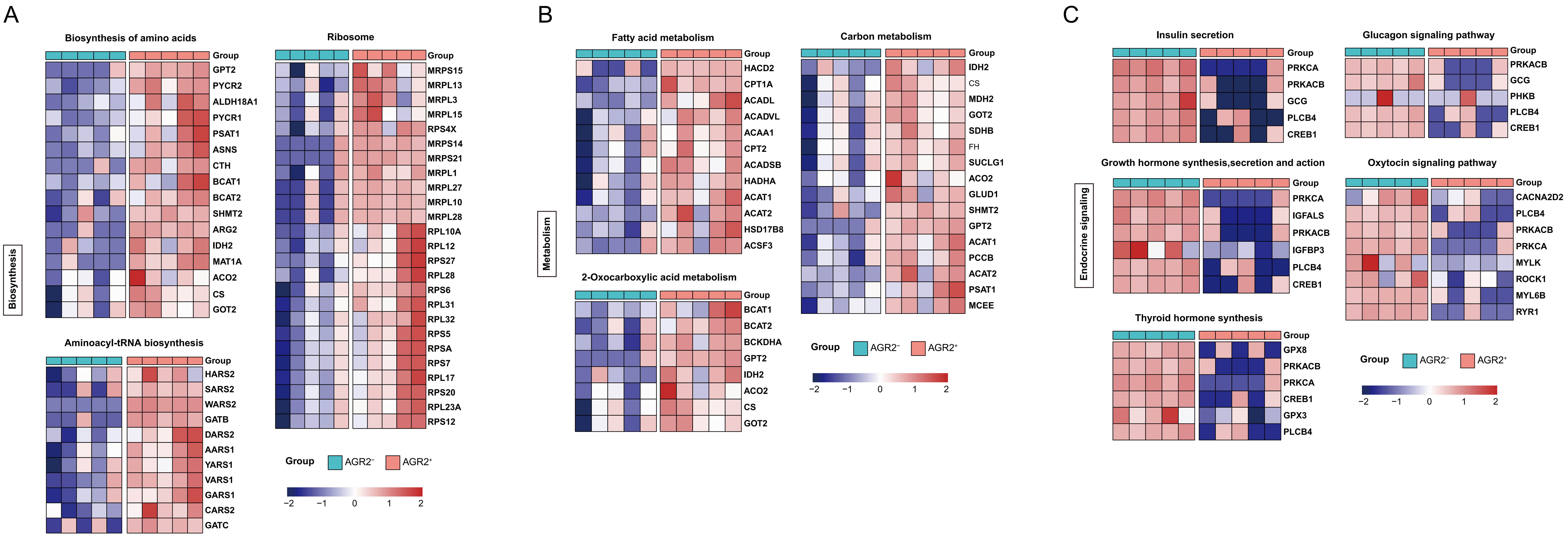

Supplementary Figure S1. Pathway-level heatmaps of representative KEGG pathways in AGR2⁺ and AGR2⁻ tumor subpopulations.
(A) Biosynthesis-related pathways, including biosynthesis of amino acids, aminoacyl-tRNA biosynthesis, and ribosome.
(B) Metabolism-related pathways, including fatty acid metabolism, carbon metabolism, and 2-oxocarboxylic acid metabolism.
(C) Endocrine signaling pathways, including insulin secretion, glucagon signaling pathway, growth hormone synthesis, secretion and action, oxytocin signaling pathway, and thyroid hormone synthesis. Columns represent patient-matched AGR2⁺ and AGR2⁻ samples, and rows represent pathway-associated proteins. Protein abundance is shown as scaled relative expression, with red indicating higher abundance and blue indicating lower abundance.
